# Ex Vivo Intestinal Organoid Models: Current State-of-the-Art and Challenges in Disease Modelling and Therapeutic Testing for Colorectal Cancer

**DOI:** 10.3390/cancers16213664

**Published:** 2024-10-30

**Authors:** Sarron Randall-Demllo, Ghanyah Al-Qadami, Anita E. Raposo, Chenkai Ma, Ilka K. Priebe, Maryam Hor, Rajvinder Singh, Kim Y. C. Fung

**Affiliations:** 1 Health and Biosecurity, Commonwealth Scientific and Industrial Research Organisation, Adelaide 5000, Australia; sarron.randall-demllo@monash.edu (S.R.-D.); ghanyah.al-qadami@csiro.au (G.A.-Q.);; 2 Health and Biosecurity, Commonwealth Scientific and Industrial Research Organisation, Westmead 2145, Australia; anita.raposo@csiro.au (A.E.R.); chenkai.ma@csiro.au (C.M.); 3 Division of Gastroenterology, Lyell McEwin Hospital, Adelaide 5112, Australia

**Keywords:** intestinal organoid, 3D culture, colorectal cancer, organ-on-a-chip, disease modelling, therapeutic screening, ex vivo models

## Abstract

Despite improvements in participation in population-based screening programme, colorectal cancer remains a major cause of cancer-related mortality worldwide. Targeted interventions are desirable to reduce the health and economic burden of this disease. Two-dimensional monolayers of colorectal cancer cell lines represent the traditional in vitro models for disease and are often used for diverse purposes, including the delineation of molecular pathways associated with disease aetiology or the gauging of drug efficacy. The lack of complexity in such models, chiefly the limited epithelial cell diversity and differentiation, attenuated mucus production, lack of microbial interactions and mechanical stresses, has driven interest in the development of more holistic and physiologically relevant in vitro model systems. In particular, established ex vivo patient-derived explant and patient-derived tumour xenograft models have been supplemented by progress in organoid and microfluidic organ-on-a-chip cultures. Here, we discuss the applicability of advanced culturing technologies, such as organoid systems, as models for colorectal cancer and for testing chemotherapeutic drug sensitivity and efficacy. We highlight current challenges associated with organoid technologies and discuss their future for more accurate disease modelling and personalized medicine.

## Simple Summary

Colorectal cancer is one of the most commonly diagnosed cancers worldwide and is the second leading cause of cancer-related deaths. In the last decade, significant advances have been made in the development of cell-based models of disease, including for culturing and expanding cells derived from patient biopsy tissue. These advances have led to the development of intestinal organoids, 3D cellular models of the colon, that more accurately represent the disease state and hold the potential to improve our understanding of disease processes. Furthermore, increasing the complexity of these models to include important components, such as the gut microbiome and the stromal compartment with immune cells and vasculature, will provide deeper insight into individual drug responses and lead to better patient outcomes in the future.

## 1. Introduction

Colorectal cancer (CRC) is the third most common cancer worldwide [1]. Despite the establishment of screening programmes in several countries and the improved management of CRC, approximately 2 million new diagnoses and 1 million deaths occurred in 2022 [1]. The increasing prevalence of CRC is mirrored by the rise in associated healthcare costs, where studies have pointed to the rising costs and healthcare burden related to the management of the disease in countries with high incidences, such as the UK, US and NZ [2,3,4,5]. Improved efficiencies in the clinical management of CRC, particularly in the prevention, detection, and treatment, hold the potential to reduce the growing burden of this disease.

In particular, treatment options for advanced CRC have grown, with newer targeted chemotherapies and immunotherapies increasing survival for patients with metastatic disease [6,7]. Examples include monoclonal antibodies such as cetuximab (Erbitux) and panitumumab (Vectibix) that target epidermal growth factor receptor (EGFR), immune checkpoint inhibitors nivolumab (Opdivo) and pembrolizumab (Keytruda) [8,9], and agents targeting vascular endothelial growth factor (VEGFR) signalling to disrupt tumour vascularisation such as bevacizumab (Avastin) and ramucirumab (Cyramza) [10]. Despite the proliferation of novel therapeutics, the clinical uptake of these specific therapies is limited by the dearth of biomarkers to direct their use in CRC. Accepted markers are few: KRAS/BRAF mutation contra-indicating the use of anti-EGFR immunotherapy and MSI-H status indicating a higher probability of response to checkpoint inhibitor therapy [11,12,13]. The majority of patients with CRC, therefore, benefit little from the emergence of new immunotherapies.

Identifying clinically beneficial therapeutics that can be directed against CRC remains challenging, with a high number of drug candidates never reaching the market. The study of the drug approval process suggests that even of those drugs reaching Phase 2 or 3 clinical trials, up to 79% fail to meet the necessary safety and efficacy profile for approval [14,15]. The likelihood of obtaining US FDA drug approval is especially low for anti-cancer therapeutics, with an overall success rate as low as 3% [16]. Contemporary strategies offered to address this lack of success relate to “fast-fail” approaches to improve selection of promising candidates, including novel computational (artificial intelligence (AI)/machine learning (ML)) approaches to drug design, identifying better surrogate markers to measure physiological endpoints, and improving the predictivity of model systems by applying ex vivo models with direct relevance to humans [17].

Since a landmark paper in 2009 which demonstrated the ability of intestinal stem cells to self-organise into viable intestinal organoids in vitro [18], organoid models have gained traction as viable preclinical models for drug screening, with the potential to augment or replace traditional 2D monolayer cultures in this setting. Organoids are 3D stem cell-derived multicellular systems that can represent a specific organ, including its cellular diversity, morphology and tissue architecture. To date, organoids have been derived to represent many organ systems, including the intestine, liver, kidney, and brain. Organoids can be derived from pluripotent stem cells or directly from tissue biopsy material (reviewed in [19]). When obtained from patients directly, these patient-derived organoid models have been shown to retain the genetic characteristics of the original tissue and are amenable to long-term culture and cryopreservation [20], representing significant technological advances for the development of personalised or precision therapies.

**Figure 1:**
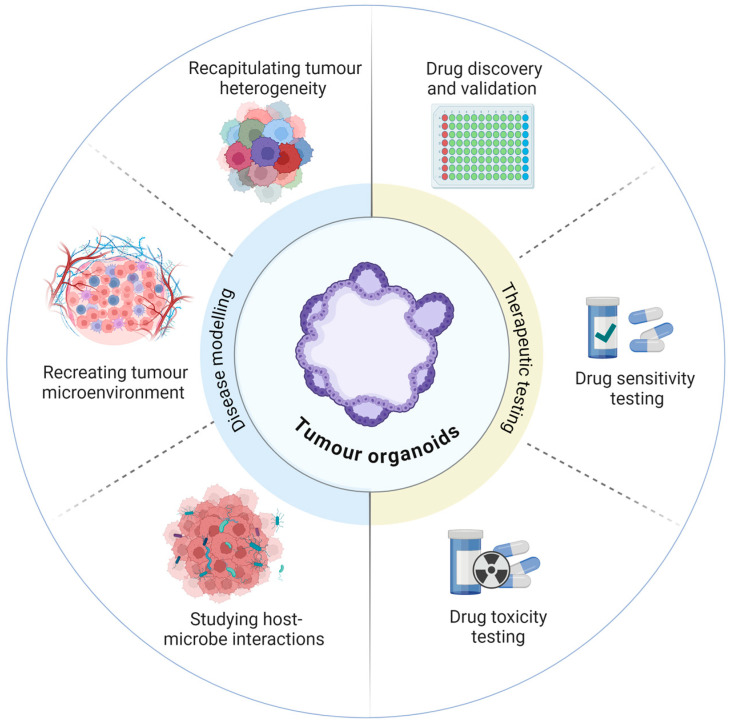
Patient-derived organoids can be applied to both disease modelling and the preclinical phases of therapeutic testing.

Incorporating phenotypic screening using ex vivo patient-derived model systems, such as patient-derived organoid (PDO), xenograft (PDX), or explant (PDE) models, into the initial stages of discovery is one approach that can potentially improve the success rate and efficiency of therapeutic drug discovery [21,22,23,24]. The choice of model system requires some consideration and varies in their fidelity to intestinal pathophysiology, with each model system naturally presenting its own unique set of limitations, e.g., heterogeneity, complexity, scalability, amenability to automation and throughput, and is summarised in Table 1. Additionally, each model system possesses its own unique advantages and limitations when applied to the drug discovery process. These are described in Table 2. Organoid models would fit best into three “domains” in the discovery and preclinical phases: early-phase target/drug discovery and validation, mechanistic studies/preclinical refinement, and preclinical efficacy/toxicity (Figure 1) and also present opportunity for capturing patient diversity at each phase [25]. While there are many publications describing in vitro models for CRC drug screening [22,26,27], this review discusses the current status and challenges associated with culturing and screening patient-derived 3D organoids as a preclinical tool for CRC disease modelling, drug evaluation and prediction of clinical outcome.

## 2. Ex Vivo Epithelial Organoids

Colonic epithelial organoids are recognised as a promising model system for studying colonic carcinogenesis; they offer a number of advantages to facilitate the identification of underlying molecular pathways and novel predictive markers of therapeutic response. When used as an adjunct to existing preclinical models of tumourigenesis, together, these can form critical points for decision-making in the research and development of new diagnostics and pharmacological therapies. Organoids can be generated from whole isolated crypts or from isolated crypt-base stem cells from the primary tissue [20]. Epithelial tissue is obtained relatively easily from mucosal biopsy during colonoscopy, from both normal and tumour tissue and organoids derived from these tissues can be perpetually expandable while retaining characteristics of the donor tissue [35]. This feature is of critical importance—while we can induce mutations in epithelial stem cells to replicate carcinogenesis, the transformation does not guarantee carcinogenesis. This is obvious when comparing precursor lesions, which may eventually give rise to cancer from disparate molecular and functional pathways. The ability to scale organoid cultures to become a source of various tumour precursor tissue through to advanced carcinoma for in vitro large-scale high-throughput screening and follow-up experimentation [20,36]. Preservation of the genomic and transcriptomic heterogeneity present in different subtypes of colonic tumours makes these organoid models highly appealing [37]. For example, it is difficult to replicate models representing the different molecular subtypes of CRC with high fidelity, such as models that have high levels of microsatellite instability that are thought to arise outside the conventional adenoma pathway and are associated with *BRAF* V600E mutations or those with defective DNA mismatch repair mechanisms, which are associated with resistance to 5-fluorouracil chemotherapy [38]. Besides differences in tumour subtypes, considerable genetic variability exists within a single tumour mass, attributable to the progressive local expansion of divergent subclones which are themselves the product of an underlying global genetic instability. For example, Roernk et al. (2018) demonstrated differential drug responses to organoids derived from different clones of the same colorectal tumour mass. Each clonal organoid consisted of a unique mutational signature that was retained during expansion [39]. Coppo et al. (2023) further demonstrated the effect of intratumour heterogeneity and derivation of clonal subpopulations on organoid growth dynamics and drug responses [40]. Truly representative organoid models which incorporate the heterogeneity present within tumours overcome known potential confounders for biomarker and drug discovery efforts, which utilise 2D in vitro cultures derived from clonally restricted cell lines.

An assembly of a “living biobank” of matching normal, precancerous and cancerous, patient-derived epithelial organoids potentially provides an immensely valuable source of tissue to support organoids for drug discovery, in particular for personalised treatments and when utilising precancerous adenomas, to test drugs at an early stage of the disease to prevent progression [34,41]. These patient-derived organoids recapitulate the heterogeneity and genetic mutations of the individuals, creating a biobank representative of the clinical heterogeneity. For example, Luo et al. (2023) established a patient-derived adenoma organoid biobank consisting of 37 organoid lines from 33 patients that was used to screen a 139 compound library identifying four compounds which consistently inhibited adenoma growth by over 50% of a bortezomib control [42]. Similarly, there are a number of reports detailing the successful establishment of colorectal tumour organoid biobanks for modelling disease subtypes and for predicting patient response to therapy [34,37,39,43,44,45,46,47,48]. Table 3 lists registered clinical trials involving patient-derived organoids to guide therapy.

The establishment of patient-derived colorectal organoids can be complicated by technical issues such as microbial contamination and unwanted mixed normal or stromal cell populations if tumour margins are not well defined. The intestinal tract contains bacteria, viruses and yeasts that may be present within mucosal tissues or the adherent mucus at the time of surgery. For example, unwanted overgrowth of microbial contamination was the cause of failure of the establishment of 21% of primary oesophageal organoid cultures in one study [49]. Bacterial and fungi may be suppressed through treatment of the starting material and initial cultures with a cocktail of antibiotics (e.g., penicillin, streptomycin, gentamycin and antifungals such as amphotericin B or nystatin) [50]. The use of metronidazole has been reported for controlling anaerobic bacteria associated with primary colonic tissues [51]. However, prolonged use of such antibiotics should be avoided since antibiotics may both inhibit cell growth and conceal low-level infection from organisms such as mycoplasma. Instead, surveillance for infection through microscopic observation, biochemical or molecular testing (e.g., qPCR, 16S sequencing), and the use of the aseptic culture technique and sterile reagents/consumables are recommended [50]. Maintaining sterile technique and adequate biological containment is also important, given the possibility of pathogenic viral infection.

Establishing organoid models from primary tissues presents the risk of culturing undesired cell populations from admixed tissues. The difficulty of obtaining organoids from epithelial tumours that are composed purely of neoplastic cells in long-term culture is a problem now highlighted by several studies of prostate and lung cancer-derived organoids [52,53,54]. Where clearly abnormal morphologies exist, morphology alone may serve as a convenient marker for the manual separation of tumour and normal organoids in conjunction with mutational analysis and copy number profiling [52,55]. Selection media deficient in selected components, such as stem cell niche growth factors normally supplied by the stroma, may provide the means to eliminate contaminating normal epithelial cells from admixed cultures. Since the majority of colorectal tumours carry mutations in the Wnt signalling pathway, removal of Wnt and R-spondin from the culture medium may reliably produce pure cultures of tumour organoids [56]. The use of selection media, however, may result in a diminished number of successfully established organoid lines, perhaps concordant with the success rate for patient-derived cell line generation [52,57]. Given the genetic heterogeneity within the neoplasms, it is unclear how many subclones may be retained through selection. More precise selection through the addition of chemical inhibitors targeting wild-type signalling pathways, such as TP53, may better preserve an organoid culture success rate suitable for large-scale screening [52].

Protocols that enable the establishment of long-term cultures have been developed with the aim of preserving the characteristics of the original tissue, summarised by Luo et. al. (2022) [58]. Supplementation of basal culture media with additives, such as small molecule inhibitors, can influence cellular behaviour (e.g., cell growth or differentiation) and gene expression patterns, potentially introducing experimental biases and impacting the reproducibility of results. Also important is the preservation of histopathological subtypes and genetic mutational profiles of the cultures for a faithful representation of the disease model [44]. For example, the addition of the p38 MAPK inhibitor SB202190 to the basal medium is regarded as essential for the proliferation of colorectal organoids but inhibits the growth of BRAF-mutated cancer organoids [20,59]. The authors demonstrated differential effects due to SB202190 on signalling pathways in or-ganoid cultures harbouring different mutational signatures. SB202190 increased EGFR phosphorylation and decreased Akt phosphorylation in a cohort of 8 cultures; only two cultures also demonstrated decreased Erk1/2 phosphorylation, as would be expected with the addition of the p38 MAPK inhibitor [59]. Similar results were observed in a second cohort of 17 cultures. NGS analysis revealed the presence of a BRAF mutation in the cultures exhibiting reduced Erk1/2 phosphorylation, including a rare active point mutation at position V471. 

## 3. Developing Model Complexity

### 3.1. Replicating the Tumour Microenvironment

To date, the majority of patient derived tumour organoid models described in the literature are composed of colonic epithelial cells and do not replicate the tumour microenvironment [60]. This includes representation of the surrounding stromal compartment such as the vasculature required to sustain the growth of tumours in vivo or the immune microenvironment that influences patient response to therapy and drug resistance. Co-culture models that include cells derived from the stromal compartment, such as cancer-associated fibroblasts and immune cells, are reviewed in Yuan et al. (2023) [61].

Due to their ability to directly affect tumour cell death, tumour-infiltrating cytotoxic T lymphocytes are of particular interest in anti-tumour responses. The frequency and activity of cytotoxic T lymphocytes isolated from the blood of patients with CRC are not ideal prognostic markers [62]. Accurate prediction of lymphocyte activity in the in vivo tumour micro-environment may be frustrated by the methods used to isolate, culture and stimulate them in vitro. Re-incorporation of infiltrating or resident immune effector cells into in vitro tumour models can assist in identifying markers of effective anti-tumour immune responses [63], studying the on-target off-tumour toxicities of immunotherapy treatments, capturing clinical toxicities not predicted by conventional tissue-based models as well as inter-patient variabilities in drug and immunotherapy responses [64]. Collection of peripheral blood mononuclear cells from donors is an ideal source of patient-matched T lymphocytes. In one study, a two-week co-culture of autologous T lymphocytes with IFN-γ-stimulated tumour organoids enabled the identification of patients whose organoids stimulated cytotoxic T lymphocytes, i.e., separation into responders and non-responders based on MHC-I status and cytolytic efficiency [63].

T lymphocytes with modified chimeric antigen receptors (CAR) have been used in haematological malignancies; however, there has been varying success in their use for other cancers. Co-culture of matched patient-derived normal and tumour organoids with CAR-engineered NK-92 cells has been reported as a sensitive in vitro platform to evaluate CAR efficacy and tumour specificity [65]. The authors noted that killing efficiency varied between organoids of different sizes, with slower kinetics observed for larger organoids, which might reflect the CAR response against solid tumour masses in vivo more closely.

Lymphocyte co-culture with healthy, non-cancerous colonic organoids has also been used to identify potential cross-reactivity in responder T cells. One consideration raised by the tumour-lymphocyte co-culture is the possibility of animal proteins in the supporting matrix, in this case, Geltrex™, an EHS sarcoma-derived hydrogel, generating cross-reactive T lymphocytes against host epithelial cells [63].

An alternative method for generating a more realistic in vitro colorectal tumour model employed a common organoid culture medium in an air-liquid interface (ALI) culture system [66]. The described ALI system preserved the stromal fibroblast and a diverse array of functional immune cells from myeloid and lymphoid lineages. Demonstrating the utility of a complex tumour model, TIL responses to PD-1/PD-1L immunotherapy could be quantified and co-cultured, though a progressive decline with repeated passage was apparent in colonic cultures.

Drug responses to anti-cancer chemotherapy are also influenced by the permeability and biochemistry of endothelium. It has been demonstrated that ex vivo models that include endothelial cells representing tumour vasculature are more predictive of drug responses than colorectal tumour organoids or 2D monolayers, highlighting the importance of the endothelium in a co-culture model. Hachey et al. (2021) showed a differential response to FOLFOX between the 2D culture of colorectal cancer cell lines (SW480 and HCT116) and 3D co-culture models of the same cell lines with the vasculature. The vasculature model showed similar drug sensitivity to xenograft tumours (1.12 ± 0.01 vs. 1.19 ± 0.27 fold change reduction in tumour size), but significantly different responses to the 2D monolayer culture, which experienced a 96% reduction in cell number [67]. Methods available to replicate angiogenesis in organoid models are reviewed in Grebenyuk and Ranga (2019) [68]. At present, many patient-derived organoid models are cultured in multi-well plates under static conditions with the establishment of tumour-vascular interactions involving the co-culture of the organoid above a monolayer of endothelial cells. One challenge associated with vascularising patient-derived organoids is their differing growth requirements and supporting ECM requirements in vitro, as recently highlighted in Rajasekar et. al. (2020) [69]. The authors determined that self-assembly of endothelial cells into microvascular structures was impeded by collagen/laminin-rich gels, such as the Matrigel favoured for colonic organoids. To compensate, a perfusion model was engineered to include a hydrogel matrix consisting of fibrin, Matrigel, and culture media that is compatible with both microvasculature and colonic organoid growth.

### 3.2. Host-Microbial Interactions

A diverse community of trillions of microorganisms resides in the mucosal surfaces of the gastrointestinal tract (GIT) from the mouth to the rectum and plays a key role in mucosal health and homeostasis. The number of microbes varies along the different segments of GIT, with the greatest number (10^10^–10^14^ CFU/mL) of these microbes residing in the colonic mucosa [70,71]. The gut microbiota, in particular, plays a major role in maintaining intestinal physiological and immune homeostasis; however, microbiota dysbiosis and enrichment of certain pathobionts have been associated with the development of intestinal and extra-intestinal disorders, including CRC [70].

The majority of evidence on the role of the microbiota on CRC is derived from association studies, and the causal link is still largely unexplained. As such, intestinal organoids provide a valuable tool to address the association-causation gap between microbes and CRC through mechanistic studies to investigate how these microorganisms could contribute to the disease. For example, multiple studies have exposed organoids to live bacteria, bacterial lysates or bacterial-derived toxins or metabolites to determine their role in CRC development. Table 4 summarises studies that have utilised intestinal organoids and bacterial co-culture, including live bacteria, bacterial-derived toxins, conditioned media, extracellular vesicles, and metabolites.

Overall, the current evidence indicates that organoids offer a promising tool for understanding the role of the microbiota in both cancer development and treatment outcomes. The most effective way to understand the full spectrum of the role of microbes is by coculturing a community of live microbes with organoids. However, the key limitation of the current organoid systems is that it does not offer the growth conditions present in the human intestine, including the oxygen gradient that supports the growth of both aerobic and anaerobic bacteria, or mechanical forces such as luminal flow or peristalsis. Currently, there are multiple attempts to overcome these limitations. Using microinjection offers a better option to co-culture anaerobic microorganisms as the organoid lumen has a lower oxygen concentration. The key challenge is that the microinjection process is time-consuming and labour-intensive and generally requires specialised equipment, which is not practical where higher throughput is desired. To automate the microinjection process, Williamson et al. (2018) established a high-throughput semi-automated microinjection system which was able to microinject around 90 organoids/h. While this offers a promising step for automating microinjection, further optimisation is needed to create a fully automated and affordable microinjection system [72]. Another way to overcome the inaccessibility of the lumen in 3D organoids is by dissociating and growing them as monolayers, which offer easier access to apical and basal surfaces, but this still requires a way to deplete oxygen on the luminal/apical side of the epithelium. Currently, there are multiple attempts to create a culture system that interfaces normoxic and hypoxic environments using culture devices of varying degrees of sophistication. One recently described method uses a colonic epithelial organoid-derived monolayer cultured on a semi-permeable insert, and the anaerobic bacteria are introduced to the apical compartment. Non-porous rubber is used to seal the apical chamber, generating a hypoxic environment while permitting oxygen perfusion into the basal chamber to maintain epithelial respiration. In this device, the epithelium showed intact polarity, as well as mucus layer and stem cell hierarchy [73]. Human enteroid-derived monolayers cultured either on Transwells or in a single fluidic-based Intestine-chip were exposed to heat-stable enterotoxin A derived from enterotoxigenic *E. coli* under three conditions: (i) static fluid, (ii) apical and basolateral flow and (iii) flow and repetitive stretch. Introducing fluid flow coincided with a shift in epithelial cell morphology from cuboidal to columnar morphology with epithelial height increasing two-fold to approximately 20 µm, and the secretion of cyclic GMP at baseline and in response to enterotoxin, highlighting the importance of mechanical stresses in mimicking normal epithelial cell function [74]. Developing engineering-based approaches to improving the growth conditions within the organoid systems will further allow for a better understanding of how these microbes collectively contribute to CRC carcinogenesis.

**Table 4:** Intestinal co-culture with bacterial species and application to colorectal cancer.

Organoid Co-Culture Models: Pro-Tumorigenesis Mechanisms			
Organoid Type and Species	Bacterial Species	Effect Shown	Reference
Human CRC organoids	Colibactin-producing *E. coli DH10B*	DNA damage (double-strand break (DSB))	[75]
Murine colon organoids	*pks+ E. coli*	DSB, genomic instability, chromosomal aberrations andgenetic mutations	[76]
Human intestinal organoids	*pks+ E. coli*	DNA damage and oncogenic mutational signatures	[77]
Human intestinal organoids	Enterotoxigenic *B. fragilis*	Did not induce a unique mutational pattern	[78]
Mouse and human colon organoids	*F. nucleatum*, *E. coli K12 strain DH10B*, *E. coli strain LF82* and *Helicobacter pylori*	*F. nucleatum* downregulated expression of DNA repair protein (NEIL2), increased the accumulation of DNA damage and production of the IL-8	[79]
Murine intestinal organoids	Bacterial lysates of wild-type *C. jejuni* (WT) or *C. jejuni* mut*cdtB*	DNA damage	[80]
Human colon organoids	*F. nucleatum* conditioned media	Increased inflammatory responses characterised by increased secretion of TNF and activation of NF-κB, p-ERK, p-CREB signalling pathways	[81]
Human intestinal organoids	*E. coli*-derived cytolethal distending toxin	DNA damage	[82]
Human intestinal organoids	*Actinomyces odontolyticus* -derived lipoteichoic acid-rich membrane vesicles	DSB	[83]
Human CRC organoids	Biliverdin, a key metabolite produced by CRC-associated *E. faecalis*	Increased the expression of cell proliferation marker Ki67	[84]
Human colon organoids	Faecal supernatant from colon cancer patients	Alterations in gene expression	[85]
Human and murine colon organoids	Faecal supernatant from a cancer mouse model lacking intestinal vitamin D receptor	Activation of JAK/STAT3 signalling and increase in PCNA and β-catenin expression	[86]
**Organoid Co-Culture Models: Protective Mechanisms**			
Murine colon organoids	*Coriobacteriaceae* (Cori.ST1911) and *Lactobacillus murinus* (La.mu730)	Upregulated expression of carnitine palmitoyltransferase 1A (CPT1A), and downregulated MUC2 protein. *Lactobacillus murinus* (La.mu730) reversed negative effect of Cori.ST1911	[87]
Human and murine CRC organoids	Short chain fatty acids	Upregulated expression of Type I IFN Stimulated Genes (CXCL10 and ISG15) which are important for anti-tumour immune response	[88]
Human CRC organoids	*Lactobacillus gallinarum* supernatant	Induction of apoptosis	[89]
Human adenoma and CRC organoids	*Lactobacillus casei*- derived ferrichrome	Tumour suppression response by upregulating the expression of DNA damage-inducible transcript 3	[90]
**Organoid Co-Culture Models: Mechanisms Related to Treatment Response**			
Murine tumour organoids	*Salmonella enterica serovar Typhimurium* (aromatase A–deficient Salmonella Typhimurium (STm^ΔaroA^)	Altered gene expression analysis including reduced expression of stem cell and EMT markers, increased expression of innate immunity proteins	[91]
Human CRC organoid	*F. nucleatum*	Enhanced efficacy of anti-PD-L1 immunotherapy	[92]

### 3.3. Intestine-on-a-Chip

Advances in tissue engineering, microfluidics and microfabrication technology have permitted the culture of human cells in an environment mimicking important aspects of anatomy and complex, dynamic physiological functions. Still, in its infancy, intestinal organ-on-a-chip models are being designed to mimic the native environment by compartmentalising the organ-specific functional components and by incorporating dynamic fluid flow to simulate blood circulation and perfusion of the intestinal lumen to support co-culture with immune cells, endothelial cells, microbes, and stromal cells [93,94,95]. These models have the potential to overcome some of the limitations of organoids, which predominantly contain the epithelial layer and an enclosed lumen, which limits access to nutrients or drugs, and does not permit mechanical stimuli to be applied [96]. Currently, the majority of organ-on-a-chip models utilises commercially available cell lines or pluripotent stem cells differentiated into multiple cell types of the intestine for proof of concept studies. This includes Caco2 or HT29 cells to study barrier integrity for drug absorption [96], organoids derived from mouse intestinal tissue [95] and human iPSCs [97]. While representing a significant improvement over standard cell lines, models derived from iPSCs typically display a foetal phenotype, failing to reach the maturity of an adult organ. Although adult stem cells (ASCs) derived from tissue biopsy can be more difficult to obtain and are limited in their ability to differentiate into non-organ specific cell types, they allow for more accurate modelling of tumour processes and provide an opportunity for personalised medicine and therapies to be explored [60,98].

Although microfluidic systems provide an ideal platform for the development of an intestine-on-a-chip that can be used for cancer modelling or drug screening applications, technical challenges still need to be overcome before they are able to be widely adopted [93]. For application as a fast-fail approach to drug discovery, i.e., deploying models with high physiological relevance earlier in the drug discovery phase, the low-throughput microfluidic devices typically used to answer research questions will need to be adapted to high-throughput screening. Scaling these low throughput systems involves surmounting key biological and engineering challenges summarised in Probst et al. (2018) [99]. Limitations, such as the requirement for specialised skills for consistent and reproducible microfabrication and use of the device and challenges associated with working with the microscale size currently limit broader adoption outside of the research community. Additionally, intestine-on-a-chip models are still limited in their ability to recreate the layers of the intestinal walls such as the smooth muscle layer required for peristalsis or the enteric nervous system, which stimulates the production of signalling molecules [96]. Technical challenges also exist for the inclusion of the microbial community into an intestine-on-a-chip platform, which is critical to recreate the true microenvironment and recapitulate the in vivo response [97]. While challenging to assemble and optimise, intestine-on-a-chip devices have uniquely enabled the in vivo-like co-culture of cancerous colonic epithelial cells (Caco-2 or organoid-derived), in normoxic media, with strictly anaerobic bacteria requiring hypoxic media (≤1% oxygen) for their growth [100,101,102]. 3D printing has offered relatively simple culture vessels that offer alternatives to the complex micro-scale systems used in some existing intestine-on-a-chip technology as it applies to CRC, such as a versatile platform with a rhomboid culture chamber to enable the growth of relatively large volumes, 0.5 cm^3^–1 cm^3^ spherical CRC microtissues with continuous perfusion with culture medium [103]. The well-characterised Caco2 cell line was used to demonstrate an extended culture of the cells within the chamber and proof-of-concept image-based on-chip assays assessing sensitivity to 5-fluorouracil. Although not suited to high-throughput screening applications, it could be used to predict tumour chemosensitivity from biopsied patient tissue. Overall, the continuous improvement of intestine-on-a-chip systems will widen the spectrum of organoid applications to address different aspects of CRC development, treatment and prevention.

## 4. Future Directions

Ex vivo technologies in cancer research, including those being developed for CRC research, encompass complex cellular models that include multiple cell types and the intestinal microbiome that are more physiologically relevant than current 2D immortalised cell lines currently in use today. While primary cell lines, PDX, and PDE are regarded as closely representative of the in vivo response, they lack key features that make 3D culture systems, such as organoid models, attractive for disease modelling and therapeutic testing. However, before organoid technology is more widely adopted for clinical use, important factors also need to be considered. These include standardisation of procedures for culturing and storage, development of robust workflows and protocols that are transferable and transparent, ethical considerations (e.g., patient informed consent), and manufacturing to scale of reagents and component parts. For research and development to progress toward real-world clinical application, regulatory frameworks need to be developed and deployed consistently across multiple jurisdictions.

In the context of CRC, intestinal organoids have the potential to improve outcomes for patients. In particular, the development of organoid biobanks with samples derived from patients has the ability to represent the heterogeneity of the disease, including the different molecular subtypes and clinical course of the disease. When compared with organoids derived from matched adjacent normal mucosa, both efficacy and toxicity of potential therapeutics can be assessed. While single organ-on-a-chip models have been reported in the literature, interconnecting these individual organs into multi-organ systems remains challenging. When multi-organ systems are combined, this replication of the “body-on-a-chip” has the potential to provide a platform to test the effect of drugs on different organs, which is an important advancement in drug discovery. Patient-specific information, such as tumour specific microenvironment, immune responses, drug metabolism and pharmacokinetics, and drug resistance mechanisms, can be more accurately assessed. Traditionally, drug efficacy and toxicity are tested in vivo using animal models, which are costly, resource-intensive, often failing to provide accurate data for human studies and present ethical arguments. As the technology develops and evolves, the organ-on-a-chip and body-on-a time and -chip concepts have the potential to revolutionise our understanding of the disease and shape clinical studies to the benefit of the patients.

## 5. Conclusions

The applications for three-dimensional culture are expanding throughout academic and industrial settings, indicating that physiological relevance is desirable despite the complexity and cost of these advanced cellular models. It should be emphasised that the field is still evolving, with fundamental challenges associated with faithfully recapitulating the intestinal organ still to be addressed, e.g., retaining the in vivo diversity of cellular types and the response to stress or biological insult. Standardisation and control over variability and the development of reproducible and validated assays applicable to 3D culture is another aspect undergoing continued development, including the use of AI for microscopy and high-content imaging. Additionally, microfluidic devices promise to provide enhanced monitoring and control over culture conditions, offering real-time, continuous and non-destructive measurements of biomarkers through arrays of biosensors. Increased development of miniaturised microfluidic systems within an SBS plate format, incorporating gravity-driven perfusion, presents an interesting compromise between complex, high-fidelity culture systems and the convenience of fitting into existing higher throughput workflows. Furthermore, research to reproducibly manufacture microfluidic devices to scale for applications beyond academic research is ongoing, and examples of commercial availability of these devices continue to grow.

As with all model systems, a balance between complexity and practicality must be struck. The use of banked, patient-derived organoids together with new “-omic” technologies to guide assays measuring biological function may significantly improve the efficiency of biomarker discovery and drug-development studies. By achieving a higher quality of putative biomarkers and drug responses from organoid-based screening, clinical validation can be initiated with greater confidence, resulting in higher success rates in human studies. Moreover, the refinement of preclinical research and development using these models will enable novel chemoprevention strategies and screening of new drugs, leading to improved patient outcomes for CRC and other intestinal diseases.

## Figures and Tables

**Table 1:** Design considerations in modelling colorectal cancer for drug discovery using patient-derived tissues.

	Primary Intestinal Cells (Transwell Support)	Patient-Derived Organoids	Organ-on-a-Chip	Patient Derived Xenografts	Patient Derived Explants
Represents in vivo system—Native organ structures?	Crypt-like formation in collagen-based gels with ALI [28,29]	“Spherical” (3D), with crypt-like formation, differentiated cell types present [20]. Preserves cell–cell interactions [30].	Organoid, Tubular (3D) and planar (2D)	“Spherical” (3D) [31]	High fidelity, overall architecture retained, mixed mucosal cell types
Preservation of Intra-tumoural heterogeneity	Low	Medium	Variable	Medium	High
Clonogenicity	High, >70% in stem cell culture, low in ALI [29]	Low, possibly <2% under differentiating conditions [29]	NA	NA	Low, differentiation and maturation close to normal
Accessible lumen	No	Yes, [32]	Organoids: no Planar/tubular cultures: yes	No	Yes
Long-term culture	Stem cells repeat passages	Yes	Possible, likely depends on ECM stability	Viability diminished after 3–5 passages	Static culture: Viability declines after 7 days medium perfusion: Bioreactor: 30 days
Throughput	Low-throughput format	Scalable, grown in multiwell format, up to 1536-well plates	Generally low to medium	Difficult to achieve—Labour and costs prohibitive even for organoid grafts	Non-scalable—limited by size of starting material
Biobanking	Stem cell banking [29,33]	High success rate, existing CRC organoid banks [34]	NA	Tumours can be banked	Can be cryopreserved but not expandable
Genetic manipulation	Yes	Yes	Yes	Direct from tumour: No Organoid grafts: Yes	No

**Table 2:** Advantages and limitations of the different preclinical models used in the drug discovery.

	Advantages	Limitations
Patient-derived organoids	Genetically and phenotypically stable over long-term culture Retains genetic background of the tumour, hence amenable to personalised medicine Able to test for drug resistance Can be scaled for medium to high throughput screen	Does not contain cellular components of the tumour microenvironment Can be expensive to establish, especially if specific growth factors are required for long term expansion Not able to predict pharmacokinetic responses
Patient derived xenografts	Able to obtain ADME data/information Allows for in vivo response	Lower throughput Expensive and resource intensive Can be difficult to establish
Patient derived explants	Provides physiological relevance and native tissue architecture Retains tumour microenvironment Cost effective and relatively easy to establish	Short duration for culturing Limited tissue viability Dependent on availability of fresh tissue
2D cancer cell line models	Amenable to high throughput screens Well established end point assays	Poor representation of tumour heterogeneity, genetic background, histological subtypes Low correlation with in vivo response Genetic heterogeneity within cell lines due to emergence of subclones

**Table 3:** Current registered clinical trials involving patient-derived organoids.

Trial Number	Study Title	Study Status	Conditions	Interventions	Country
NCT05669586	Organoids Predict Therapeutic Response in Patients With Multi-line Drug-resistant Lung Cancer	Recruiting	Lung Cancer	Phase 2	China
NCT04768270	The Culture of Ovarian Cancer Organoids and Drug Screening	Recruiting	Ovarian Cancer	Observational, patient registry	China
NCT05092009	Lung Cancer Organoids and Patient Derived tumour Xenografts	Recruiting	Lung Cancer	Observational	The Netherlands
NCT05290961	The Culture of Advanced or Recurrent Ovarian Cancer Organoids and Drug Screening	Recruiting	Ovarian Neoplasms	Observational, patient registry	China
NCT06064682	An Organoid-based Functional Precision Medicine Trial in Osteosarcoma	Recruiting	Osteosarcoma	Observational, standard of care biopsy	USA
NCT05577689	Novel Therapy Target in Metastatic Prostate Cancer	Not yet recruiting	Prostate Neoplasms	Observational	China
NCT05832398	Precision Chemotherapy Based on Organoid Drug Sensitivity for Colorectal Cancer	Recruiting	Colorectal Cancer	Interventional	China
NCT04931394	Organoid-Guided Adjuvant Chemotherapy for Pancreatic Cancer	Recruiting	Pancreatic Cancer	Interventional, phase 3	China
NCT04931381	Organoid-Guided Chemotherapy for Advanced Pancreatic Cancer	Recruiting	Advanced Pancreatic Cancer	Interventional, phase 3	China
NCT06268652	Patient Derived Organoid-guided Personalised Treatment versus Treatment of Physician’s Choice in Breast Cancer	Recruiting	Breast Cancer, Refractory Breast Carcinoma	Interventional, phase 3	China
NCT05024734	Guiding Instillation in Non Muscle-invasive Bladder Cancer Based on Drug Screens in Patient Derived Organoids	Recruiting	Bladder Cancer, Non-muscle Invasive	Interventional, phase 2	Switzerland
NCT05725200	Study to Investigate Outcome of Individualised Treatment in Patients With Metastatic Colorectal Cancer	Recruiting	Metastatic Colorectal Cancer	Interventional, phase 2	Norway
NCT06468527	Clinical Trial to Evaluate the Efficacy and Safety of Dirocaftor/Posenacaftor/Nesolicaftor in Adults With CF	Recruiting	Cystic Fibrosis	Interventional, phase 2	The Netherlands
NCT06102824	Organoid-based Functional Precision Therapy for Advanced Breast Cancer	Recruiting	HER2-negative Breast Cancer, Advanced Breast Cancer	Interventional, phase 2	China
NCT05352165	The Clinical Efficacy of Drug Sensitive Neoadjuvant Chemotherapy Based on Organoid versus Traditional Neoadjuvant Chemotherapy in Advanced Rectal Cancer	Not yet recruiting	Neoadjuvant Therapy	Interventional	China
NCT06227065	Precise Neoadjuvant Chemoresection of Low Grade NMIBC	Not yet recruiting	Bladder Cancer, Non-muscle Invasive Bladder Cancer	Interventional, phase 2	Switzerland
NCT03979170	Patient-derived Organoids of Lung Cancer to Test Drug Response	Recruiting	Lung Cancer	Observational, patient registry	Switzerland
NCT03283527	Chemoradioresistance in Prospectively Isolated Cancer Stem Cells in Esophageal Cancer-Organoid: RARE STEM-Organoid	Recruiting	Esophageal Cancer	Observational	The Netherlands
387579 (ACTRN12624000684527p)	FORECAST-II Feasibility of using Organoid Response to inform treatments for patients with Colorectal cancer staring first-line therapy	Not yet recruiting	Colorectal Cancer	Diagnosis/ prognosis	Australia
386544 (ACTRN12623001136695)	ORganoId GuIded N-of-1 (ORIGIN-1) Trial: A phase 4 study to investigate whether people with cystic fibrosis (CF) with rare cystic fibrosis transmembrane regulator (CFTR) mutations who have an in vitro response to Trikafta will also have a clinically meaningful response to Trikafta versus placebo	Not yet recruiting	Cystic Fibrosis	Interventional, phase 4	Australia
380279 (ACTRN12620001353987)	FORECAST 1. Feasibility of using Organoid Response to find Effective Treatments for patients with Colorectal cancer After failure of Standard Therapy	Recruitment closed	Metastatic Colorectal Cancer	Interventional	Australia
NCT03544255	Drug Screening of Pancreatic Cancer Organoids Developed From EUS-FNA Guided Biopsy Tissues	Unknown status	Pancreatic Cancer	Observational	China
NCT03544047	Clinical Study on Drug Sensitivity Verification or Prediction of Therapy for Breast Cancer by Patient-Derived Organoid Model	Unknown status	Breast Cancer	Interventional	China

References: https://anzctr.org.au/TrialSearch.aspx; https://clinicaltrials.gov/, accessed on 5 September 2024.
